# The Combination Therapy of Dietary Galacto-Oligosaccharides With Budesonide Reduces Pulmonary Th2 Driving Mediators and Mast Cell Degranulation in a Murine Model of House Dust Mite Induced Asthma

**DOI:** 10.3389/fimmu.2018.02419

**Published:** 2018-10-23

**Authors:** Kim A. T. Verheijden, Saskia Braber, Thea Leusink-Muis, Prescilla V. Jeurink, Suzan Thijssen, Aletta D. Kraneveld, Johan Garssen, Gert Folkerts, Linette E. M. Willemsen

**Affiliations:** ^1^Division of Pharmacology, Faculty of Science, Utrecht Institute for Pharmaceutical Sciences, Utrecht University, Utrecht, Netherlands; ^2^Department of Nephrology, Radboud Institute of Molecular Life Sciences, Radboud University Medical Center, Nijmegen, Netherlands; ^3^Division of Veterinary Pharmacy, Pharmacology and Toxicology, Faculty of Veterinary Sciences, Utrecht University, Utrecht, Netherlands; ^4^Nutricia Research, Immunology, Utrecht, Netherlands

**Keywords:** allergy, asthma, house dust mite, galacto-oligosaccharides, budesonide

## Abstract

**Background:** Dietary non-digestible galacto-oligosaccharides (GOS) suppress allergic responses in mice sensitized and challenged with house dust mite (HDM). Budesonide is the standard therapy for allergic asthma in humans but is not always completely effective.

**Aim:** To compare the efficacy of budesonide or different doses of GOS alone or with a combination therapy of budesonide and GOS on HDM-allergic responses in mice.

**Methods:**BALB/c mice were sensitized and challenged with HDM, while fed a control diet or a diet supplemented with 1 or 2.5 w/w% GOS, and either or not oropharyngeally instilled with budesonide. Systemic and local inflammatory markers, such as mucosal mast cell protease-1 (mMCP-1) in serum, pulmonary CCL17, CCL22, and IL-33 concentrations and inflammatory cell influx in the bronchoalveolar lavage fluid (BALF) were determined.

**Results:** Budesonide or GOS alone suppressed the number of eosinophils in the BALF of HDM allergic mice whereas budesonide either or not combined with GOS lowered both eosinophil and lymphocyte numbers in the BALF of HDM-allergic mice. Both 1 w/w% and 2.5 w/w% GOS and/or budesonide suppressed serum mMCP-1 concentrations. However, budesonide nor GOS alone was capable of reducing Th2 driving chemokines CCL17, CCL22 and IL-33 protein levels in supernatants of lung homogenates of HDM allergic mice, whereas the combination therapy did. Moreover, IL-13 concentrations were only significantly suppressed in mice treated with budesonide while fed GOS. A similar tendency was observed for the frequency of GATA3^+^CD4^+^ Th2 and CD4^+^RORγt^+^ Th17 cells in the lungs of the allergic mice.

**Conclusion:** Dietary intervention using GOS may be a novel way to further improve the efficacy of anti-inflammatory drug therapy in allergic asthma by lowering Th2 driving mediators and mast cell degranulation.

## Introduction

Asthma is a chronic disease affecting 235 million people worldwide. The disease is characterized by airway hyperresponsiveness, airway narrowing and airway inflammation containing high numbers of eosinophils (1, 2). House dust mite (HDM) is a common allergen that can induce allergic diseases like asthma and allergic rhinitis (3). Different cytokines (e.g., IL-33 and GM-CSF) and chemokines (e.g., CCL20) can be released by airway epithelial cells after stimulation with HDM contributing to allergic sensitization (4). IL-33 and GM-CSF are capable of activating dendritic cells (DC) and group 2 innate lymphoid cells (ILC2), which produce IL-5 and IL-13 (2, 5). Furthermore, immature DC are attracted to the lung by CCL20, and in turn are activated by Th2 driving mediators (6). After activation, DC release chemokines such as CCL17 and CCL22 known to drive the development of Th2 effector responses from naïve T cells in the mediastinal lymph nodes. These Th2 cells will migrate to the pulmonary mucosa and like ILC2 produce IL-13 and drive allergic sensitization and symptoms of allergic airway inflammation (4, 6, 7). Long-acting beta agonists with or without glucocorticosteroids are the most commonly used drugs for asthma. Glucocorticosteroids, such as budesonide, are known to bind and activate the intracellular glucocorticoid receptor, which translocates into the nucleus and suppresses the transcription of pro-inflammatory genes, while enhancing transcription of certain regulatory genes of activated immune cells and structural cells (8). The glucocorticosteroids are added to suppress the ongoing allergic airway inflammation. Nevertheless, when the drug is discontinued the effects of inhaled corticosteroids rapidly disappear. However, long-term treatment with glucocorticosteroids, even in low concentrations, can have considerable side effects, such as weight gain, reduced growth in children, and muscle weakness (9–11). Current treatment is therefore still not sufficient and novel preventive and/or therapeutic approaches are needed for asthmatic disorders. The gut microbiota has a substantial influence on the systemic immune function. Different animal and human studies indicated changes in the intestinal microbiota may contribute to development of asthma (12–15). Specific non-digestible oligosaccharides such as galacto-oligosaccharides (GOS) are selectively fermented in the intestine resulting in support of growth and/or activity of bifidobacteria and lactobacilli (12, 16, 17). Non-digestible oligosaccharides can have a preventive effect on allergic diseases. In different murine ovalbumin (OVA) asthma models a combination of GOS/long-chain (lc) fructo-oligosaccharides (FOS)/pectin-derived acidic oligosaccharides (AOS) suppressed airway inflammation and hyperreactivity (18, 19). Furthermore, GOS suppressed allergic airway eosinophilia in ovalbumin-sensitized rats (20). Clinical studies indicated that a mixture of 9:1 GOS/lcFOS provided in the first year of life still has a protective effect against allergic manifestations at 5 years of age for allergic rhinoconjunctivits and atopic dermatitis (21). In addition, GOS/lcFOS in combination with *Bifidobacterium breve* reduced atopic dermatitis scores in young infants when given for a period of 12 weeks, Interestingly. later in life these children experienced less wheezing and coughing apart from cold and had reduced requirements for asthma medication compared to the control group (22). In adult patients suffering from asthma, this dietary intervention reduced Th2 cytokine production and increased peak expiratory flow (23). In the present study, the effectiveness of combined dietary intervention with GOS and with budesonide, a gold standard reference treatment, on pulmonary inflammation and mast cell degranulation was investigated in a murine HDM-induced allergic asthma model.

## Methods

### Mice

Male BALB/c mice (Charles River, Maastricht, The Netherlands), 6 to 8-week old were housed under bio-contained sterile conditions using HEPA® filtered isocages® (Tecniplast, Buguggiate, Italy). Food and water were provided *ad libitum*. All animal experiments were conducted in compliance with the Guidelines of the Ethical Committee on the Use of Laboratory Animals of the Utrecht University (DEC 2013.II.08.090).

### HDM murine asthma model

**Figure 1:**
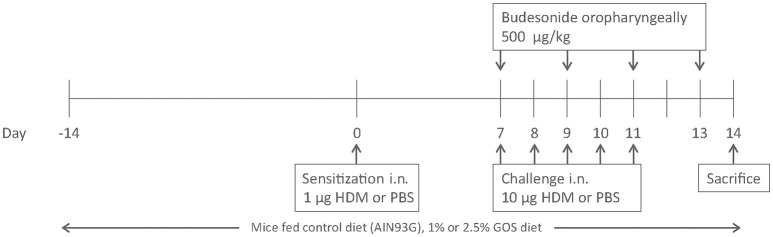
Induction of house dust mite allergy in mice. BALB/c mice were intranasally (i.n.) sensitized with PBS or house dust mite (HDM) on day 0 and challenged on days 7 to 11 intranasally with PBS or HDM. Mice were fed control diet (AIN93G, contr) or 1 w/w% or 2.5 w/w% GOS from day −14 to 14 and either or not oropharyngeally instilled with budesonide on days 7, 9, 11, and 13. All mice were sacrificed on day 14.

While under isoflurane anesthesia, BALB/c mice were intranasally (i.n.) sensitized with PBS in presence or absence of 1 μg HDM (Greer Laboratories, Lenoir, USA) and challenged i.n. with PBS or 10 μg HDM on days 7 to 11 while being fed a diet (AIN-93G, control diet) containing 0, 1 or 2.5 w/w% GOS (Vivinal® GOS syrup with approximately 59% galacto-oligosaccharides, 21% lactose, 19% glucose, and 1% galactose on dry matter (dry matter of 75%); FrieslandCampina Domo, Zwolle, The Netherlands). Carbohydrates in Vivinal® GOS were compensated isocalorically in the control diet by means of exchange against cellulose (for GOS), lactose (for lactose), and dextrose (for glucose), from day−14 to day 14 (24). On day 7, 9 and 11 budesonide (500 μg/kg, Sigma-Aldrich, Zwijndrecht, The Netherlands) was either or not instilled oropharyngeally, after induction of a light isoflurane anesthesia, 6 h prior to the daily challenge and day 13, 24 h prior to sacrifice on day 14 (25, 26) (Figure 1).

### Airway responsiveness measurement

The EMKA invasive measurement of dynamic resistance (EMKA Technologies, Paris, France) in response to increasing doses of methacholine (acetyl-β-methyl-choline chloride, Sigma-Aldrich) (0–25 mg/ mL, 10% puff for 10 s) was used to measure lung function in anesthetized mice. Data are presented as average lung resistance (RL) in cm H2O/mL^*^sec-1 (26).

### Serum preparation

After sacrifice, blood samples were collected by cardiac puncture. The blood was coagulated 30 min at room temperature and centrifuged at 14,000 rpm for 10 min. Serum samples were stored at −20°C until further use.

### ELISA HDM-specific IgE

Elisa plates (Corning 9018) were coated overnight with HDM (50 μg/mL in PBS), washed, blocked for 1 h with a 0.1% w/v BSA in PBS, washed and incubated with 5x diluted serum samples. Plates were washed and incubated for 1.5 h with 1 μg/mL biotin anti-mouse IgE (553419, BD Biosciences), washed and incubated for 1 h with streptavidin-HRP (Sanquin, Amsterdam) containing ELISA buffer, washed and incubated with TMB (ready to use, eBioscience) and 2M H_2_SO_4_ was used to stop the reaction. The absorbance was measured with the iMark microplate reader (Bio-Rad) at 450 nm (27).

### Bronchoalveolar lavage

Lungs were lavaged with 1 mL of pyrogen-free saline (0.9% NaCl, 37°C) supplemented with protease inhibitor cocktail tablet (Complete Mini, Roche Diagnostics, Mannheim, Germany). This was followed by 3 lavages with 1 mL saline solution (0.9% NaCl, 37°C). The BALF cells were centrifuged (400 g, 5 min) and pellets of the lavages were pooled and total numbers of BALF cells were counted using a Bürker-Türk chamber (magnification 100x). For differential BAL cell counts, cytospin preparations were made and stained with Diff-Quick (Merz and Dade A.G., Düdingen, Switzerland). Numbers of lymphocytes, eosinophils, macrophages and neutrophils were scored with light microscopy (28). Afterwards the lavage the right lung was used to prepare lung homogenates, while half of the left lung was used for flowcytometry and the other half of the left lung for *in vitro* re-stimulation.

### Preparation of lung homogenates

Lung samples were homogenized in 1% Triton X100 (Sigma-Aldrich)/PBS containing protease inhibitor (Complete Mini, Roche Diagnostics) using a Precellys 24 tissue homogenizer (Bertin Technologies, Toulouse, France). Homogenates were centrifuged at 14,000 rpm for 10 min, supernatants were collected and stored at −20°C until further use. The protein concentration was measured using the Pierce BCA protein assay kit standardized to BSA according to the manufacturer's protocol (Thermo Fisher Scientific, Rockford, USA). The homogenates were diluted to a final concentration of 1 mg protein/mL prior to cytokine or chemokine measurements (29, 30).

### Lung restimulation with house dust mite *ex vivo*

Lung cell suspensions were prepared after enzymatic digestion of the lungs using digestion buffer, containing DNase I and Collagenase A (Roche Diagnostics), for 30 min. The digestion was stopped by adding fetal calf serum (FCS, Hyclone Laboratories, Logan, USA). The lung pieces were passed through a 70 μm filter and rinsed with 10 mL RPMI. Cells were washed and resuspended in RPMI 1640 culture medium (Lonza, Allendale, USA) supplemented with 10% heat-inactivated FCS and 0.1% penicillin-streptomycin solution (Sigma-Aldrich). Lung cells (4 × 10^5^ cells/well) were cultured in medium with or without 50 μg/mL HDM (Greer Laboratories). The supernatant was harvested after 4 days of culture at 37°C in 5 % CO_2_ and stored at −20°C until further analysis (31).

### Lung T cell subsets assessed by flow cytometry

Aspecific background was blocked using PBS blocking buffer containing 1% BSA and 5% FCS for 30 min. 5 × 10^5^ cells were plated per well and incubated at 4°C for 30 min with different antibodies against CD4-PerCP Cy5 (cat no. 45-0042, clone RM4-5), CD69-FITC (cat no. 11-0691, clone H1.2F3), GATA3-PE (cat no. 12-9966, clone TWAJ), Tbet-eFLUOR660 (cat no. 50-5825, clone eBio4D10), RORγt-PE (cat no. 12-6988, clone AFKJs-9), Foxp3-APC cat no: 17-5773, clone FJK-16s (eBioscience, San Diego, USA), CD8a-APC Cy7 (cat no. 557654, clone 53-6.7) and CD25-FITC (cat no. 553071, clone 7D4CD25 FITC) (BD, Breda, The Netherlands) and matching isotype controls were used. Cells were permeabilized for intracellular staining using fixation/ permeabilization buffer set, according to manufacturer's protocol (eBioscience). Flow cytometry was conducted using FACS Canto II (BD) and analyzed using Flowlogic Software (Inivai Technologies, Victoria, Australia) (32).

### Measurement of cytokines and chemokines

IL-33, GM-CSF, CCL17, CCL20, and CCL22 were measured with a DuoSet ELISA (R&D Systems, Abingdon, United Kingdom), IL-13, IL-5 and mMCP-1 with a Ready-SET-Go!® ELISA (eBioscience) all according to manufacturer's protocol. Cytokine concentrations in supernatants of lung cell restimulation were determined by a standard IL-13 flex set (BD Biosciences). The concentrations of these mediators were expressed as pg/mg protein in supernatants of lung homogenates and pg/mL in restimulation supernatants and serum.

### Histology

Lungs were fixed with 10% formalin via a cannula inserted in the trachea at a constant pressure of 25 cm H_2_O. After 24 h of fixation the lungs were embedded in paraffin and 5 μm sections were cut. Sections were stained with hematoxylin and eosin, according to standard methods. Photomicrographs were taken with an Olympus BX50 microscope equipped with a Leica DFC 320 digital camera, using 200X magnification. Three slides per animal were reviewed in blinded fashion by two independent observers and the percentage of surface area that was infiltrated with inflammatory cells was scored as follow: score 0 no inflammation (0%), score 1 mild inflammation (>0–<30%), score 2 moderate inflammation (>30–<60%), score 3 severe inflammation (>60–100%) (33).

### Statistical analysis

Data are presented as mean ± standard error of mean (SEM). Data were statistically analyzed using a one-way ANOVA followed by a Bonferroni's multiple comparisons test. *P* < 0.05 were considered significant. Statistical analyses were conducted using GraphPad Prism software (version 6.04).

## Results

### Dietary intervention with GOS in combination with budesonide is most effective in reducing eosinophil numbers in BAL-fluid

**Figure 2:**
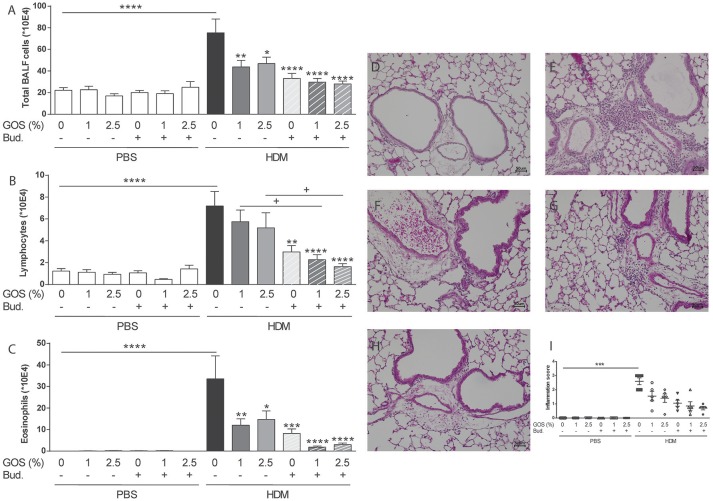
Combination of dietary GOS and budesonide effectively reduces eosinophilic inflammation in the lungs of HDM-allergic mice. Inflammatory cell influx in the BALF of house dust mite allergic mice. Total BAL cells **(A)**, absolute number of lymphocytes **(B)**, eosinophils **(C)**. Results are shown as mean ± SEM. Statistical significance of differences was tested using *post hoc* Bonferroni's multiple comparisons test after One-Way ANOVA. ^*^P < 0.05, **P < 0.01, ***P < 0.001, ****P < 0.0001 compared to the HDM-contr group, + *P* < 0.05 *n* = 8–9 mice/group. Representative photomicrographs of the lungs stained with H&E. PBS- control diet **(D)**, HDM-control diet **(E)**, HDM- control diet and budesonide treatment **(F)**, HDM-1 w/w% GOS diet **(G)**, HDM-1 w/w% GOS diet and budesonide treatment **(H)**, inflammation score of histological photomicrographs; the percentage of tissue surface area that was infiltrated with inflammatory cells was scored blinded as follows: score 0 no inflammation (0%), score 1 mild inflammation (>0– < 30%), score 2 moderate inflammation (>30– < 60%), score 3 severe inflammation (>60–100%) **(I)**. Results are shown as mean ± SEM. Statistical significance of differences was tested using Kruskall Wallis test. Magnification 200x, BALF *n* = 8–9 mice per group, histology *n* = 5 mice/group. PBS, PBS- sensitized and PBS- challenged mice (white bars); HDM, HDM -sensitized and challenged mice (gray bars); Contr, control diet; GOS, 1 w/w% GOS or 2.5 w/w% GOS diet; Bud, budesonide.

To study the extent of airway inflammation in HDM-allergic mice upon dietary intervention with 1 w/w% or 2.5 w/w% GOS or intra-airway treatment with budesonide or a combination, the bronchoalveolar lavage was examined. The total number of inflammatory cells was significantly increased in the HDM mice compared to the PBS group both fed the control diet (Figure 2A) This was due to a significant increase in the number of lymphocytes and eosinophils (Figures 2B,C). Dietary intervention with 1 w/w% or 2.5 w/w% GOS significantly reduced the number of total BAL cells and eosinophils, whereas treatment with budesonide reduced total BAL cells, eosinophils, and lymphocyte numbers. When GOS and budesonide were combined the amount of eosinophils almost returned to baseline levels (Figure 2C). An increase of inflammatory cells was also observed in lung histology sections of the HDM mice compared to the PBS mice fed the control diet (Figures 2D–I). Dietary intervention with 1 w/w% or 2.5 w/w% GOS, treatment with budesonide or the combination of both showed a similar decreasing pattern in inflammation score (Figures 2D–I). In addition to pulmonary inflammation, the airway resistance was measured to investigate the lung function in HDM asthmatic mice. There were no differences between the experimental groups on basal level. Methacholine dose-dependently increased airway resistance and a significant increase in lung resistance (6.25–12.5 mg/mL of methacholine) was observed in HDM mice fed the control diet compared to the PBS control group. The GOS diet did not affect this, whereas budesonide alone showed a strong tendency to prevent the increase in airway resistance compared to HDM allergic mice fed the control diet which became significant when budesonide treated HDM allergic mice were also fed the 1 or 2.5% GOS diet in the groups exposed to 6.25 or 12.50 mg/mL methacholine (Supplementary Figure 1). The number of macrophages or neutrophils was not affected upon HDM challenge or not significantly affected by the treatments (Supplementary Figures 2A,B).

### Dietary intervention with GOS in combination with budesonide decreases serum mMCP-1 and chemokine and cytokine concentrations in lung homogenates

**Figure 3:**
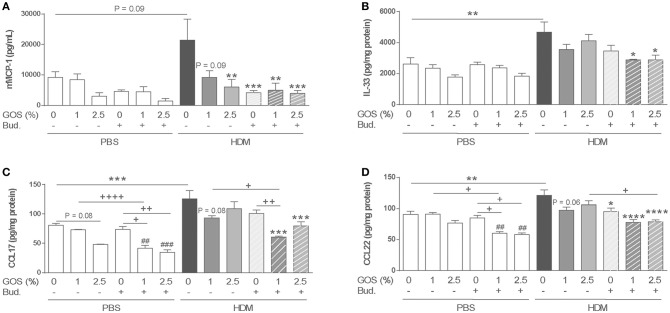
Combination of dietary GOS and budesonide suppresses mMCP-1 serum concentration and Th2 driving mediators in lung homogenates of HDM allergic mice. mMCP-1 **(A)** (pg/mL in serum) and IL-33 **(B)**, CCL17 **(C)**, CCL22 **(D)** (pg/mg protein in supernatant of lung homogenates) concentrations were measured. Statistical significance of differences was tested using *post hoc* Bonferroni's multiple comparisons test after One-Way ANOVA. ^*^P < 0.05, **P < 0.01, ***P < 0.001, ****P < 0.0001 compared to the HDM-contr group, +*P* < 0.05, ++*P* < 0.01, + + ++*P* < 0.0001, and ##*P* < 0.01, ###*P* < 0.001 compared to the PBS-contr group, *n* = 6 mice/group. Results are shown as mean ± SEM. PBS: PBS–sensitized and–challenged mice (white bars), HDM, HDM-sensitized and–challenged mice (gray bars). Contr, control diet; GOS, 1 w/w% GOS or 2.5 w/w% GOS diet; Bud, budesonide. Results are shown as mean ± SEM.

To examine the effect on mast cell degranulation, mMCP-1 concentrations were measured in serum. The expression of mMCP-1 in the HDM control group tended to increase compared to the PBS control group. Dietary intervention with 1 w/w% GOS or 2.5 w/w% GOS, as well as budesonide treatment or the combination of both, significantly decreased the levels of mMCP-1 (Figure 3A). In HDM-allergic mice HDM-IgE levels were increased compared to control mice, however these were not significantly affected by the treatments (Supplementary Figure 3A). IL-33 was significantly increased in supernatants of lung homogenates of HDM allergic mice. Dietary GOS or budesonide treatment did not affect this, while the combination significantly decreased IL-33 concentrations in HDM allergic mice (Figure 3B). The combination therapy decreased basal CCL17 and CCL22 concentrations already in control animals (Figures 3C,D). In HDM-allergic mice, CCL17 and CCL22 were significantly enhanced and again only the combination therapy significantly decreased these concentrations (Figures 3C,D). GM-CSF and CCL20 did not significantly increase in HDM allergic mice compared control mice these (data not shown).

### Dietary intervention with GOS in combination with budesonide decreases pulmonary Il-13 concentrations

**Figure 4:**
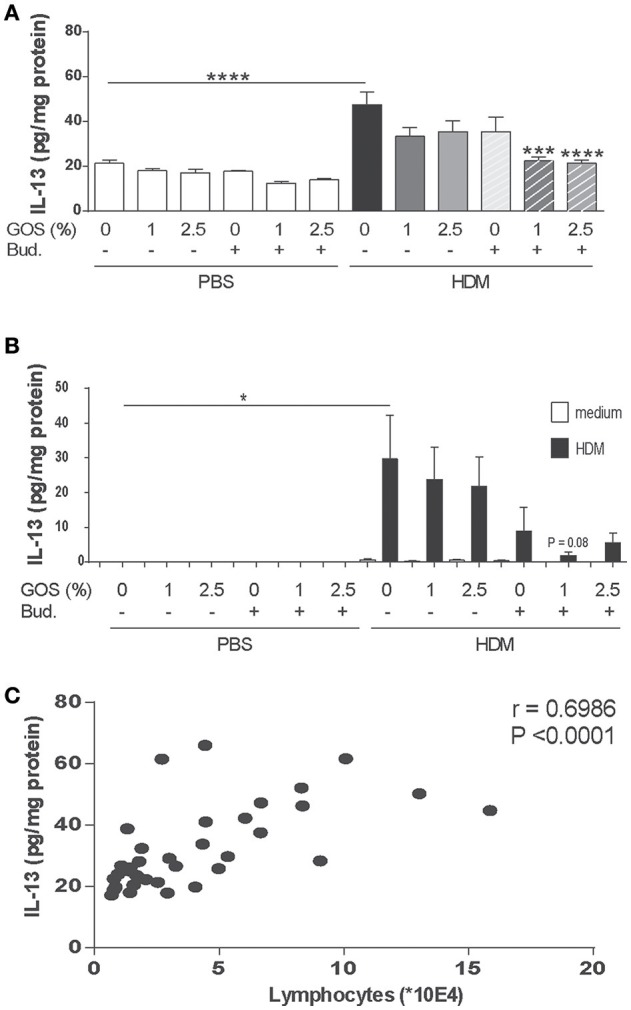
Combination of dietary GOS and budesonide reduces IL-13 concentrations in the lungs of HDM-allergic mice. IL-13 concentrations were measured in supernatants of lung homogenates (pg/mg protein) **(A)** and in supernatants of lung cell suspensions upon *ex vivo* HDM restimulation (pg/mL) **(B)**. Correlation of IL-13 concentration in lung homogenates and the number of lymphocytes between the HDM-groups **(C)**. Results are shown as mean ± SEM. Statistical significance of differences was tested using *post hoc* Bonferroni's multiple comparisons test after One-Way ANOVA. ^*^P < 0.05, ***P < 0.001, ****P < 0.0001 compared to the HDM-contr group *n* = 6 mice/group. Correlation was assessed using the Spearman correlation test. PBS, PBS–sensitized and–challenged mice (white bars); HDM, HDM-sensitized and–challenged mice (gray bars). Contr, control diet; GOS, 1 w/w% GOS or 2.5 w/w% GOS diet; Bud, budesonide.

Th2 type inflammation marker IL-13 was measured in lung homogenate supernatants. IL-13 concentrations were significantly increased in the HDM group fed the control diet (Figure 4A). This was confirmed after *ex vivo* re-stimulation of lung cell suspensions with HDM (Figure 4B). Only in budesonide treated mice fed the GOS diet, IL-13 concentrations were significantly reduced and the same tendency was shown upon HDM re-stimulation of lung cells (Figures 4A,B). IL-13 concentrations in lung homogenates of HDM mice correlated positively with the number of lymphocytes measured in the BALF (Figure 4C). IL-5 concentrations did not significantly increase in HDM allergic mice compared control mice these (data not shown).

### Decreased frequency of activated T-helper cells, Th2 and Th17 cells after dietary intervention with GOS combined with budesonide

**Figure 5:**
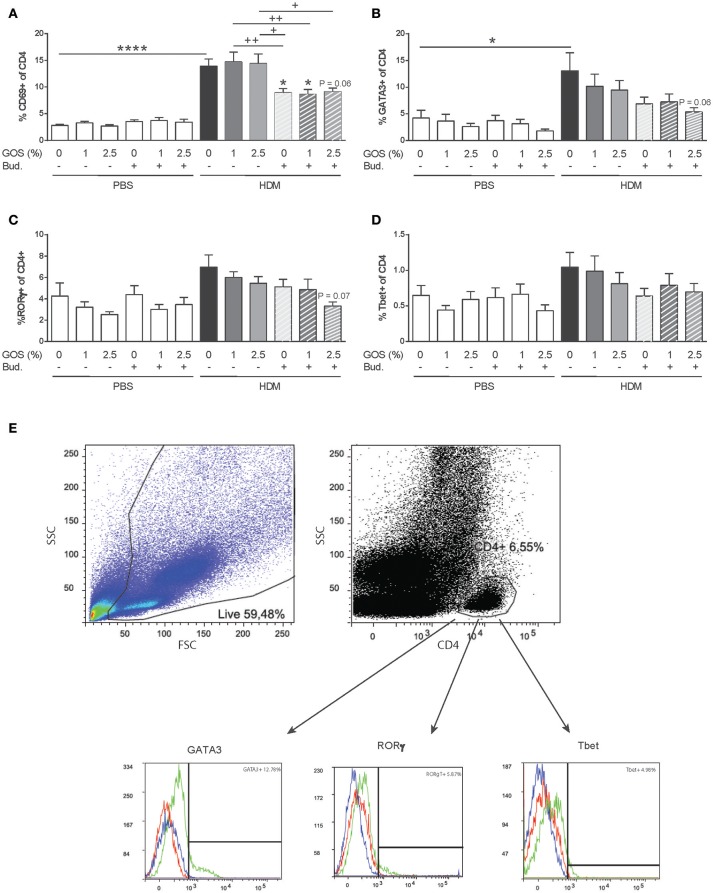
The frequency of activated Th cells and Th2 and Th17 cells decreases after dietary intervention with 2.5 w/w% GOS combined with budesonide. Representative dot plots and histograms of gating strategy of lung T helper cell subsets **(E)**. Lymphocytes were gated based on FSC-SSC pattern, and T helper cells were gated based on expression of CD4. Within the CD4^+^ population the frequency of GATA3 (Th2 cells), RORγt (Th17 cells) and Tbet (Th1 cells) was analyzed. In the histogram the blue line represents FMO control, red line isotype control and green line MFI of the specific antibody. Percentage of activated CD4^+^ cells **(A)**, GATA3^+^ of CD4^+^ cells **(B)**, RORγt^+^ of CD4^+^ cells **(C)**, and Tbet^+^ of CD4^+^ cells **(D)** was calculated. Results are shown as mean ± SEM. Statistical significance of differences was tested using *post hoc* Bonferroni's multiple comparisons test after One-Way ANOVA. ^*^P < 0.05, ^****^P < 0.0001 compared to the HDM-contr group, +*P* < 0.05, ++*P* < 0.01, *n* = 6 mice/group.

The expression of the early activation marker CD69 was increased in CD4^+^ T-helper (Th) cells in lungs of HDM mice and not affected by the GOS diet. After treatment with budesonide with or without GOS, a decreased frequency of activated Th cells was observed (Figure 5A). The frequency of GATA3^+^CD4^+^ Th2 cells was significantly increased in HDM allergic mice fed the control diet compared to the PBS control group (Figures 5B,C). The combination of GOS with budesonide tended to decrease GATA3^+^CD4^+^ Th2 cells (*p* = 0.06) (Figure 5B). Although the frequency of CD4^+^RORγt^+^ Th17 cells or CD4^+^Tbet^+^ Th1 cells was not significantly increased in HDM allergic mice compared to PBS control mice, a tendency toward a decrease in Th17 cell frequency was observed only when mice were treated with 2.5 w/w% GOS combined with budesonide (*p* = 0.07) (Figures 5C–E). The frequency of FoxP3^+^CD25^+^ of CD4^+^ Treg cells was not affected by the GOS diet in control and HDM allergic mice, but tended to be lower in the HDM allergic mice treated with budesonide compared to HDM allergic mice, in presence or absence of the GOS diet. This reached significance in the budesonide treated HDM allergic mice fed the 2.5% GOS diet (*p* < 0.05) compared to HDM allergic mice fed the 2.5% GOS diet (Supplementary Figure 3B).

## Discussion

**Figure 6:**
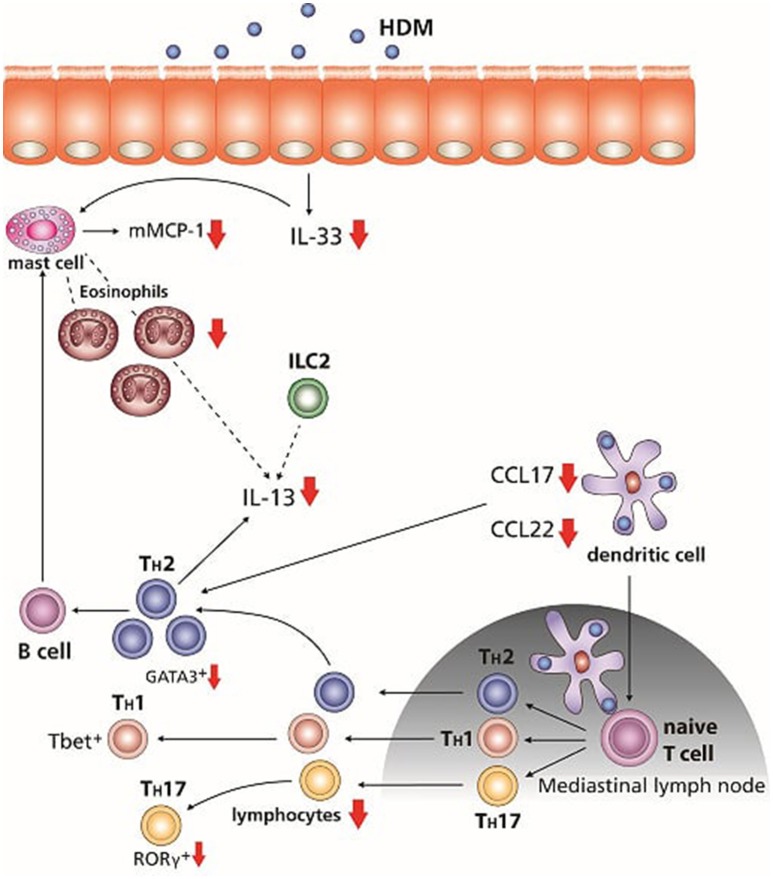
Overview of the effects of the dietary intervention with GOS combined with glucocorticosteroid budesonide treatment. After the initial exposure to HDM mMCP-1 is released by mast cells, and IL-33 is known to be secreted by the airway epithelium, which can also activate mast cells as well as DC and ILC2. Combination of the GOS diet with budesonide treatment reduced mMCP-1 and IL-33 concentrations. CCL17 and CCL22 are secreted by activated DC, which can differentiate naïve T cells into Th2 cells and are chemo-attractants for Th2 cells. The combination therapy significant suppressed the production of both chemokines. Th2 cells as well as ILC2 and mast cells are able to produce IL-13. Concentrations of IL-13 were reduced after the treatment of both GOS and budesonide. The number of lymphocytes and eosinophils was decreased by treatment with budesonide, whereas GOS alone suppressed the number of eosinophils. The combination of dietary GOS with budesonide treatment effectively suppressed both leukocyte subtypes. Only dietary intervention with GOS in combination with budesonide tended to suppress the Th2 and Th17 frequency in lung cell suspensions. Small arrows, tendency to reduce; big arrows, significant reduced.

This study was performed to examine whether a combination therapy consisting of dietary GOS with budesonide was more effective than either of the treatments alone. Dietary intervention with GOS improved the effectiveness of budesonide therapy in most features of the HDM asthma model. Murine HDM allergic asthma models are commonly used to mimic some of the human features of asthma as they show airway inflammation and pulmonary cytokine release similar to humans (34, 35). Previously we showed that GOS had similar preventive effects as budesonide in suppressing allergic features in a murine model for HDM-induced allergic asthma (26). In the current study the combination of GOS and budesonide almost completely abolished eosinophil and lymphocyte numbers, which was associated with improved airway resistance, lower inflammatory mediator release and reduced mast cell degranulation (serum mMCP-1). mMCP-1 is released by mast cells in the tissue upon allergen challenge, becomes available locally and as a reflection can be measured back in the serum (36–38) (Figure 6).

In the airways of asthmatic patients the level of IL-33, mainly expressed by bronchial epithelial cells, is increased (39, 40). After allergen stimulation, IL-33 can activate mast cells, ILC2, and dendritic cells which are driven to produce Th2 polarizing chemokines CCL17 and CCL22. Moreover, IL-33 acts as a chemo-attractant for Th2 cells (41–45). In airway tissue of asthmatics, mast cell numbers and levels of mast cell proteases are increased and also an increase in activation and recruitment of Th2 lymphocytes is observed (46). Interestingly, the IL-33 and mucosal mast cell derived mMCP-1 concentrations were significantly suppressed by the combination therapy of GOS with budesonide in HDM-HDM mice (Figure 6). The latter is of particular importance, since in asthmatic patients, budesonide had no effect on IL-33 levels (47). As IL-33 is also important for activation of Th2 driving DC and ILC2, effective reduction may result in decreased allergic inflammation and symptoms. Increased levels of CCL17 were found in BALF, plasma/serum and sputum of patients with asthma (48–50). DC, that drive allergic sensitization, produce CCL17 as well as CCL22, which are both CCR4 ligands (51) and involved in Th2 polarization, allergic sensitization and allergic effector responses as CCR4 is expressed by allergen induced Th2 lymphocytes which are attracted than to the airways in humans (52–55). Furthermore, in a murine asthma model, CCL22 or CCL17 neutralization decreased eosinophilic airway inflammation and ameliorated allergic symptoms (56). Dietary intervention with 1 w/w% GOS tended to reduce both CCL17 and CCL22, while budesonide reduced CCL22. By contrast, the combination of the GOS diet and budesonide significantly reduced the concentrations of both chemokines (Figure 6). This indicates that dietary GOS facilitates budesonide treatment in its capacity to further decrease allergy driving mediators IL-33, CCL17 and CCL22, derived from both airway epithelial cells and DC. As far as we know, such a mechanism has not been described before and may be part of the mechanism by which dietary GOS enforces the actions of budesonide treatment.

To our knowledge this study is the first to demonstrate that dietary adjunct therapy may support the anti-inflammatory properties of glucocorticosteroid treatment. Anti-inflammatory therapies still have considerable side-effects (9–11) and decreasing the dose of glucocorticosteroid may have beneficial effects for asthmatic patients. It would be interesting to know whether GOS can affect these side effects and whether lower doses of glucocorticosteroids can be used in combination with GOS in BALB/c mice as well as other mice strains like C57/BL6. In addition, the effects of the dietary intervention and/or budesonide treatment on intestinal and pulmonary microbiome composition and bacterial fermentation products should be studied, since the microbiome composition and activity has been shown affect the susceptibility to develop airway inflammation and microbial dysbiosis is observed in people affected with asthma (57–59).

T lymphocytes are important in the development of asthma. Activated T helper cells are increased in asthmatic mice (60). In the current study, budesonide treatment alone or in combination with a GOS diet showed a decrease in activated T-cell frequency (Figure 6). The reduction in Th2 driving mediators by budesonide treatment in GOS fed HDM mice may have resulted in a reduced Th2 polarization and Th2 cell influx. Indeed Th2 type cytokine concentrations of IL-13 were significantly lower in HDM allergic mice with the combination therapy (Figure 6). Antigen-specific Th2 cells, ILC2s and mast cells secrete IL-13 (41, 61, 62). In the HDM allergic mice IL-13 was not only increased in lung tissue, but also in lung cell suspensions exposed to HDM. Similar, in mild asthmatics, an increase in IL-13 mRNA levels was found in the BALF upon allergen challenge (63). GATA3 is necessary for differentiation of naive T cells into Th2 cells but also for the development and function of ILC2 (61, 64). In asthmatic patients, the GATA3 expression in T cells was five times higher compared to healthy controls whereas also bronchial epithelial cells express high levels of GATA3 (65). Here we shows that the combination therapy resulted in a tendency toward a decrease in the frequency of GATA3^+^CD4^+^ Th2 cells in the lung. As GATA3 is known to play a crucial role in the production of IL-13 by Th2 cells (61), the reduced lL-13 may be explained, at least in part, by this effect on Th2 cells (Figure 6). Beyond this effect on Th2 cell frequency, the combination therapy also tended to decrease the percentage of RORγt^+^ CD4^+^ Th17 cells (66), known to be increased in asthmatic patients (67). HDM sensitization and challenge did not affect the frequency of Treg and budesonide treatment tended to lower the %Treg which could not be rescued by the dietary intervention with GOS. However, in a previous study GOS was shown to improve the regulatory function of the CD25+ cells, which include Treg and via this way GOS may have supported the anti-inflammatory capacities of budesonide treatment (68).

In conclusion, a combination therapy of budesonide together with a dietary intervention with GOS, most effectively suppressed the allergic inflammatory response when compared to either of the treatments alone. Hence, dietary adjunct therapy using GOS may be a novel way to further improve the effectiveness of glucocorticosteroids in asthma.

## Author contributions

KV and LW designed the study, performed data analyses, and wrote the manuscript. AK and GF designed the study, helped critically discussing the data and writing the manuscript. PJ and JG were involved in critically discussing the data. KV performed the studies and SB, TL-M, and ST helped out with technical assistance and helpful discussions.

### Conflict of interest statement

KV and SB were given grants by the Carbohydrate Competence Center (CCC); PJ and JG are employees of Nutricia Research, which is an industrial partner in the Dutch Carbohydrate Competence Center. The remaining authors declare that the research was conducted in the absence of any commercial or financial relationships that could be construed as a potential conflict of interest.

## Supplementary material

Supplementary Figure 1 Pulmonary resistance upon methacholine exposure. Methacholine induced lung resistance was measured by means of EMKA and significantly increased in the HDM allergic mice at 6.25–12.5 mg/mL. This was not affected by the GOS, whereas budesonide alone tended to prevent the increase in airway resistance. Budesonide treatment of HDM allergic mice fed the 1% or 2.5% GOS significantly reduced lung resistance. Results are shown as mean ± SEM. Statistical significance of differences was tested using a One-Way ANOVA with *post hoc* Bonferroni's multiple comparisons test.0.05 ^*^*P* < (compared to HDM-control), ++*P* < 0.01 *n* = 8–9 mice/group.

Supplementary Figure 2 Effect of dietary GOS and/or budesonide on the number of macrophages and neutrophils in the BALF. The absolute number of macrophages **(A)** and neutrophils **(B)** in the BALF. Results are shown as mean ± SEM. Statistical significance of differences was tested using a One-Way ANOVA with *post hoc* Bonferroni's multiple comparisons test. ^*^*P* < 0.05, ++*P* < 0.01 *n* = 8–9 mice/group.

Supplementary Figure 3 HDM specific IgE in serum and the frequency of regulatory T-cells in lung tissue. HDM-IgE was measured in serum by means of ELISA and increased in HDM-allergic mice compared to PBS controls **(A)**. Budesonide treatment and/or the GOS diet did not significantly affect HDM-IgE levels. Within the CD4+ population the frequency (%) of Foxp3+CD25+ Treg cells was analyzed in lung tissue **(B)**. Budesonide tended to lower %Treg, which was not affected by the GOS diets. Results are shown as mean ± SEM. Statistical significance of differences was tested using a One-Way ANOVA with *post hoc* Bonferroni's multiple comparisons test. ^*^ or + *P* < 0.05, *n* = 7–13 for HDM-IgE and *n* = 8–9 for Treg mice/group.

## Abbreviations

BALF: bronchoalveolar lavage fluid
DC: dendritic cell
FOS: fructo-oligosaccharide
GOS: galacto-oligosaccharide
HDM: house dust mite
ILC2: group 2 innate lymphoid cell
OVA: ovalbumin.
