# Supplementary figures and images for: The Combination Therapy of Dietary Galacto-Oligosaccharides With Budesonide Reduces Pulmonary Th2 Driving Mediators and Mast Cell Degranulation in a Murine Model of House Dust Mite Induced Asthma
Source: Front Immunol. 2018 Oct 23;9:2419. doi: 10.3389/fimmu.2018.02419 (PMC6207001; doi:10.3389/fimmu.2018.02419)

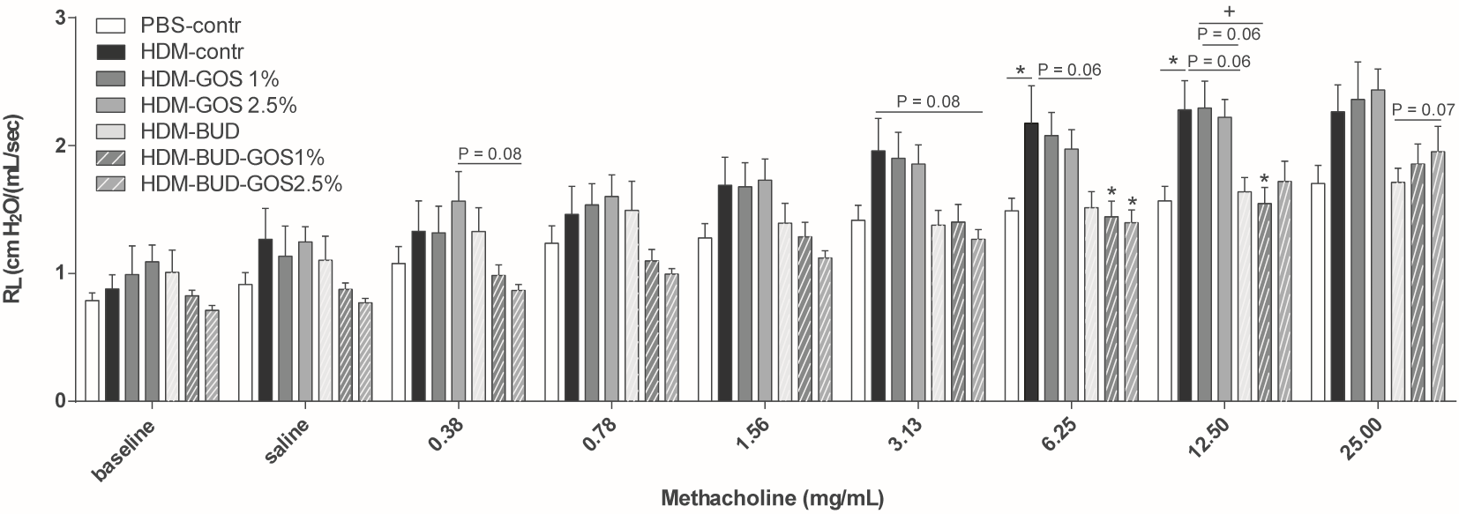

Supplement: Supplementary Figure 1 — Pulmonary resistance upon methacholine exposure. Methacholine induced lung resistance was measured by means of EMKA and significantly increased in the HDM allergic mice at 6.25–12.5 mg/mL. This was not affected by the GOS, whereas budesonide alone tended to prevent the increase in airway resistance. Budesonide treatment of HDM allergic mice fed the 1% or 2.5% GOS significantly reduced lung resistance. Results are shown as mean ± SEM. Statistical significance of differences was tested using a One-Way ANOVA with post hoc Bonferroni's multiple comparisons test.0.05 *P < (compared to HDM-control), ++P < 0.01 n = 8–9 mice/group. [file Image_1.TIF]

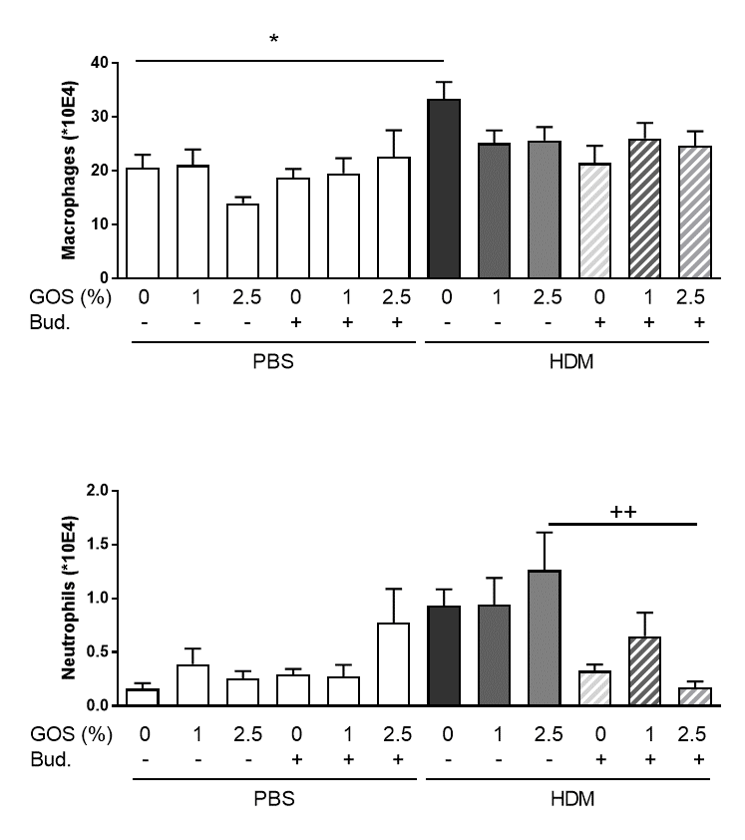

Supplement: Supplementary Figure 2 — Effect of dietary GOS and/or budesonide on the number of macrophages and neutrophils in the BALF. The absolute number of macrophages (A) and neutrophils (B) in the BALF. Results are shown as mean ± SEM. Statistical significance of differences was tested using a One-Way ANOVA with post hoc Bonferroni's multiple comparisons test. *P < 0.05, ++P < 0.01 n = 8–9 mice/group. [file Image_2.TIF]

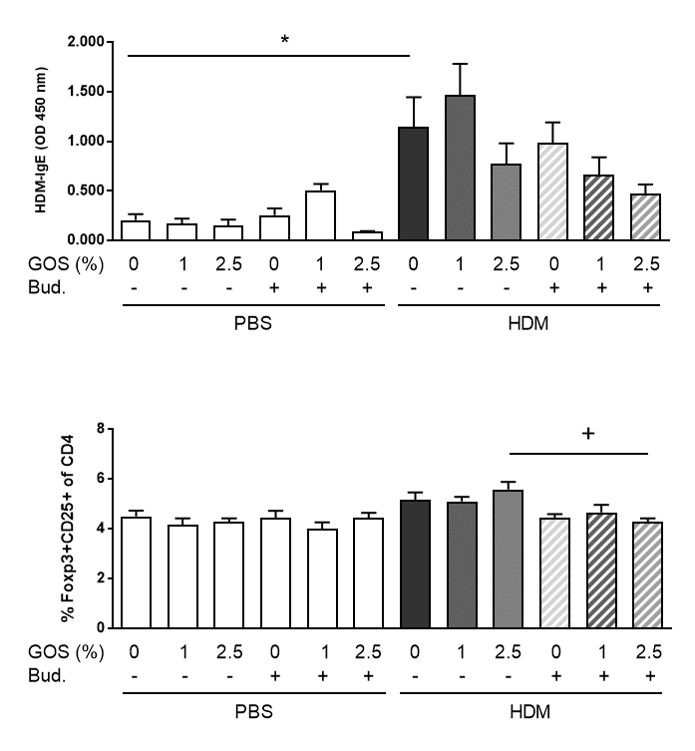

Supplement: Supplementary Figure 3 — HDM specific IgE in serum and the frequency of regulatory T-cells in lung tissue. HDM-IgE was measured in serum by means of ELISA and increased in HDM-allergic mice compared to PBS controls (A). Budesonide treatment and/or the GOS diet did not significantly affect HDM-IgE levels. Within the CD4+ population the frequency (%) of Foxp3+CD25+ Treg cells was analyzed in lung tissue (B). Budesonide tended to lower %Treg, which was not affected by the GOS diets. Results are shown as mean ± SEM. Statistical significance of differences was tested using a One-Way ANOVA with post hoc Bonferroni's multiple comparisons test. * or + P < 0.05, n = 7–13 for HDM-IgE and n = 8–9 for Treg mice/group. [file Image_3.TIF]
